# Placental exosomes in preeclampsia: unresolved questions in functional signaling and clinical translation

**DOI:** 10.1097/MS9.0000000000005207

**Published:** 2026-06-09

**Authors:** Arisha Masood, Zobia Aamir, Ayesha Irfan, Sijan Poudel

**Affiliations:** a Department of Medicine, Jinnah Sindh Medical University, Karachi, Pakistan; b Department of Medicine, Dow University of Health Sciences, Karachi, Pakistan; c Department of Medicine, Lumbini Medical College & Teaching Hospital, Pravas, Palpa, Nepal

**Keywords:** biomarkers, endothelial dysfunction, placental exosomes, preeclampsia, pregnancy

## Abstract

Preeclampsia is a hypertension condition unique to pregnancy that has a substantial global impact on maternal and fetal morbidity and mortality and is characterized by new-onset hypertension and proteinuria. The condition is caused by unusual placentation, leading to placental hypoxia and systemic endothelial dysfunction. Early prediction and effective targeted interventions are still limited. Exosomes and other extracellular vesicles are mediators of feto-maternal communication. These vesicles carry proteins, microRNAs, and other nucleic acids, detectable in maternal circulation. Total and placenta-derived exosomes are increased prior to the clinical onset of preeclampsia. Exosomal release caused by low oxygen levels leads to endothelial dysfunction and inflammatory signaling. Research focuses on exosome concentration instead of the functional impact of their molecular cargo. The specific effects of exosomes on maternal and fetal organ systems are not well understood. Future research should understand the systemic effects of exosomal communication and provide high-resolution analysis of its constituents. Applying research findings to clinical settings may allow earlier, non-invasive risk assessment and enhance outcomes in preeclampsia.

Preeclampsia, a multisystem hypertensive condition, is among the most dreaded pregnancy consequences and frequently manifests in the third trimester as proteinuria and new-onset hypertension. It accounts for about 70 000 maternal deaths and 500 000 fetal deaths annually worldwide, while it affects only 5%–7% of pregnant women and has the highest morbidity and mortality[1]. It is mainly due to abnormal placentation, which causes significant dysfunction of the endothelium and placental hypoxia because of inadequate trophoblast invasion and spiral artery remodeling. Even with extensive research, early predictions and targeted interventions are not possible. This highlights the importance and need for mechanical insights and biomarkers[2].

It has been proposed that extracellular vesicles, which are lipid-bilayer-contained structures that hold macromolecules like proteins, miRNA, and other significant nucleotides, are crucial for this feto-maternal communication. These small vesicles can be seen in the mother’s blood and other body fluids and tell us a lot about the placental milieu[3]. They are necessary for normal placental development, angiogenesis, endothelial signaling, and are both proinflammatory and immunosuppressive[4].

During pregnancy, the levels of total exosomes and placenta-derived exosomes in maternal plasma are significantly elevated in presymptomatic women who subsequently develop preeclampsia compared to controls[5]. Hypoxia-induced exosomal release plays a role in inflammatory signaling and endothelial dysfunction, which are two important aspects of placental exosomes[2].

Although progress has been made, there are still significant gaps. Most research focuses on alterations in the concentration of exosomes, but the specific function of exosomal cargo, especially molecular signatures linked to disease, is still not well understood. Furthermore, we are uncertain about the impact of exosomes on specific organs, including maternal organs and fetal organ development. Because of these gaps, we have a limited understanding of the underlying mechanism, and it is difficult to apply findings to clinical practice.

Future research should focus on understanding the systemic effects of exosomal communication and provide a more detailed, high-resolution analysis of its constituents. Moreover, it is important to apply research findings to clinical settings to enable earlier non-invasive risk assessment and improve outcomes in preeclampsia.

## Data Availability

No new data were generated or analyzed for this study. Data sharing is not applicable to this article.

## Research registration unique identifying number (UIN)

Not applicable, as this submission is a letter to the editor and does not involve primary research.

## Data availability statement

No new data were generated or analyzed for this work.

## AI usage

During the preparation of this work, the authors used ChatGPT and Grammarly to rectify grammar and spelling mistakes, check reference suitability, and tighten language. After using these tools, the authors reviewed and edited the content as needed and took full responsibility for the content of the publication.
